# Current Cigarette Smoking Among Workers in Accommodation and Food Services — United States, 2011–2013

**DOI:** 10.15585/mmwr.mm6429a5

**Published:** 2015-07-31

**Authors:** Girija Syamlal, Ahmed Jamal, Jacek M. Mazurek

**Affiliations:** 1 Division of Respiratory Disease Studies, National Institute for Occupational Safety and Health, CDC; 2 Office on Smoking and Health, National Center for Chronic Disease Prevention and Health Promotion, CDC

Tobacco use is the leading cause of preventable disease and death in the United States (1). One of the *Healthy People 2020* objectives calls for reducing the proportion of U.S. adults who smoke cigarettes to ≤12% (objective TU-1.1) (2). Despite progress in reducing smoking prevalence over the past several decades, nearly one in five U.S. adults, including millions of workers, still smoke cigarettes (1,3). During 2004–2010, nearly one fifth (19.6%) of U.S. working adults aged ≥18 years smoked cigarettes, and of all the industry sectors, current smoking prevalence among the accommodation and food services sector workers (30%) was the highest (3). CDC analyzed National Health Interview Survey (NHIS) data for 2011–2013 to estimate current cigarette smoking prevalence among adults working in the accommodation and food services sector, and found that these workers had higher cigarette smoking prevalence (25.9%) than all other workers (17.3%). Among workers in accommodation and food services sector, the highest smoking prevalences were observed among males, non-Hispanic whites, those aged 25–44 years, those with a high school diploma or a General Educational Development (GED) certificate and no college education, those with an annual family income <$35,000, those with no health insurance, and those working in the food services and drinking places industry. These results indicate a need to better understand the reasons for higher smoking prevalence observed among accommodation and food services workers (e.g., workplace culture), so that appropriate intervention strategies can be developed and implemented. Evidence suggests that smoke-free worksites and workplace cessation programs, including comprehensive worksite smoke-free policies, health promotion, access to smoking cessation programs, and increasing the cost of tobacco products, can substantially reduce smoking among workers (1,4,5).

NHIS data are collected annually from a nationally representative sample of the noninstitutionalized U.S. civilian population through a personal household interview. During 2011–2013, survey response rates ranged from 66.3% (2011) to 61.2% (2013) (6). To improve the precision and reliability of the estimates, CDC combined 2011–2013 NHIS data. Data were adjusted for nonresponse and weighted to be nationally representative; 95% confidence intervals were calculated. Two-tailed t-tests were used to determine statistically significant differences between point estimates.* Cigarette smokers were defined as adults aged ≥18 years who reported having smoked ≥100 cigarettes during their lifetime and, at the time of interview, reported smoking every day or some days. Survey participants were considered to be working currently if, when asked about their employment status during the week before their interview, they responded “working at a job or business,” “with a job or business but not at work,” or “working, but not for pay, at a family-owned job or business.” Information on participants’ industry of employment and occupation was classified by trained coders (6). Workers within the accommodation and food services sector were identified, and two industries and 10 occupations were examined within the sector.† Cigarette smoking prevalences were calculated for the accommodation and food services sector workers and for all other workers (i.e., those not working in the accommodation and food services) by selected characteristics.

During 2011–2013, approximately 142 million (60.5%) of the estimated 235 million U.S. adults aged ≥18 years were employed during the week before the interview; among these, an estimated 9.3 million (6.6%) worked in the accommodation and food services sector. Overall cigarette smoking prevalence was 17.8% among U.S. working adults, with a prevalence of 25.9% among the accommodation and food services sector workers and 17.3% among all other U.S. workers. Among the accommodation and food services sector workers, cigarette smoking was highest among those aged 25–44 years (31.4%), those with a high school diploma/GED certificate and no college education (32.1%), those with an annual family income <$35,000 (30.9%), and those with no health insurance (29.3%) (Table 1).

Compared with all other working adults who currently smoked, accommodation and food services workers who smoked were less educated (41.2% versus 36.0% with only a high school diploma/GED certificate), more likely to live below the federal poverty level (22.3% versus 11.6%), to have ≤5 years on the job (75.5% versus 58.3%), and to smoke ≥12 cigarettes per day (70% versus 61.6%) (Table 2).

Cigarette smoking prevalence among workers in the food services and drinking places (26.8%) industry and in five of the 10 occupations within the accommodation and food services sector was greater than twice the *Healthy People 2020* target of ≤12% for U.S. adults. Cigarette smoking prevalence exceeded 25% among workers in the motor vehicle operators and material moving and other transportation (37.2%), management (32.6%), supervisors of food preparers (27.3%), food and beverage servers (27.0%), and cooks and food preparers (26.5%) occupations (Table 3).

## Discussion

The U.S. Surgeon General’s report on the health consequences of smoking concluded that disease and death from tobacco use are overwhelmingly caused by cigarettes and other combustible tobacco products, and that rapid elimination of their use will substantially reduce this burden (1). Furthermore, smoking costs an estimated annual >$130 billion in direct medical expenses, $151 billion in lost productivity, and $5.6 billion for lost productivity attributable to premature deaths caused by exposure to secondhand smoke (1). This report indicates that 2.4 million workers in the accommodation and food services sector currently smoke cigarettes, and among those, prevalence was highest among males, non-Hispanic whites, persons with less education, those who live below the poverty level, those who have been working for <5 years, and those who had no health insurance. Furthermore, no significant changes in cigarette smoking prevalence were observed among accommodation and food services sector workers since 2004–2010 (3), and smoking prevalence in this sector remains significantly higher than workers in all other sectors.

Several intervention and prevention measures have been shown to be effective in reducing smoking prevalence and secondhand smoke exposure (1,4), including smoke-free workplace policies. Although workplace policies or exposures to secondhand smoke in the workplace were not assessed in this study, historical data have shown that only 43% of workers in food preparation and service occupations are covered under smoke-free worksite policies (7). Although such policies have been shown to be beneficial in reducing smoking rates, increasing quit rates among those who smoke, reducing secondhand smoke exposure among nonsmokers and thus improving overall health of workers, they have not yet been universally adopted or implemented.§

Other proven population-based interventions include increasing tobacco prices, implementing comprehensive smoke-free policies in workplaces and public places, employing anti-tobacco mass media campaigns, and ensuring barrier-free access to quitting assistance (8). Furthermore, in concert with Total Worker Health,¶ a strategy that integrates occupational safety and health protection with health promotion to prevent worker injury and illness, employers may adopt workplace interventions that address health risks from both the work environment and from individual behavior, with the goal of reducing smoking-related disparities. Employers, businesses, trade associations, and worker representatives can work in partnership with their state and local health departments to implement these evidence-based policies and programs to reduce the prevalence of smoking among U.S. workers.

**Summary**

**What is already known on this topic?**

Despite progress in reducing smoking prevalence over the past several decades, millions of working adults still smoke cigarettes, the most commonly used tobacco product in the United States. During 2004–2010, 19.6% of U.S. working adults were cigarette smokers. Among them, workers in the accommodation and food services sector had one of the highest smoking prevalences (30.0%).

**What is added by this report?**

During 2011–2013, accommodation and food services workers had nearly 50% higher smoking prevalences than all other U.S. workers, and no significant changes in cigarette smoking prevalence were observed among these workers since 2004–2010. Workers in the accommodation and food service industries and in most occupations within the sector had high smoking prevalences, which were greater than the target of *Healthy People 2020* target of ≤12% for U.S. adults.

**What are the implications for public health practice?**

Continued implementation of effective public health interventions and adoption of integrated approaches to address health risks from both the work environment and individual behavior can reduce smoking-related disparities. Employers, businesses, trade associations, and worker representatives can work in partnership with their state and local health departments in implementing evidence-based policies and programs to reduce the prevalence of smoking among the working population.

The findings in this report are subject to at least two limitations. First, the employment information collected applied only to the week preceding the interview. Some workers might have changed jobs and thus might have been in a different occupation or industry before the time of the survey. However, additional analyses examining longest held job showed similar results. Second, the extent of underreporting or over reporting of cigarette smoking could not be determined because smoking information was self-reported and was not validated by biochemical tests; nevertheless, comparison of self-reported smoking status with results of measured serum cotinine levels suggests generally high levels of validity (9).

Workers in the accommodation and food services sector have a higher prevalence of cigarette smoking than all other civilian U.S. working adults. A *Healthy People 2020* objective (TU-13) calls for all states to enact laws on smoke-free indoor air that prohibits smoking in public places and worksites (2). Although considerable progress has been made during the past decade, with increasing numbers of states having comprehensive smoke-free laws that prohibit smoking in all indoor areas of worksites, restaurants, and bars, an estimated 2.4 million workers in the food and accommodation services sector still smoke cigarettes (10). Continued adoption of proven population-based interventions, in concert with intensified implementation of comprehensive smoke-free laws in indoor public places and worksites, can reduce cigarette smoking and exposure to secondhand smoke and thus can improve individual health (1).

## Figures and Tables

**TABLE 1:** Cigarette smoking* prevalence among adults aged ≥18 years currently working† in accommodation and food services sector, by selected characteristics — National Health Interview Survey, 2011–2013

	Accommodation and food services sector workers			All non-accommodation and food services sector workers			
			
Characteristic	Estimated population§ (in thousands)	Smoking prevalence (%)	(95% CI)	Estimated population (in thousands)	Smoking prevalence (%)	(95% CI)	p value†
**Total**	**9,345**	**25.9**	**(24.3–27.4)**	**130,115**	**17.3**	**(16.9–17.8)**	**<0.001**
**Age group (yrs)**							
18–24	3,211	20.5	(17.5–23.4)	13,841	17.8	(16.4–19.3)	0.113
25–44	3,983	31.4	(28.7–34.1)	57,246	18.6	(17.9–19.2)	<0.001
45–64	1,939	25.2	(21.7–28.7)	52,652	17.0	(16.4–17. 7)	<0.001
≥65	212	11.3	(4.9–17.7)	6,376	7.7	(6.6–8.8)	0.275
**Sex**							
Male	4,323	28.3	(25.8–30.9)	69,630	19.5	(18.8–20.1)	<0.001
Female	5,022	23.8	(21.7–25.8)	60,485	14.9	(14.3–15.4)	<0.001
**Race/Ethnicity**							
Hispanic	2,338	11.1	(9.1–13.0)	19,074	13.1	(12.2–14.0)	0.066
White, non-Hispanic	5,180	33.9	(31.6–36.3)	88,880	19.0	(18.4–19.6)	<0.001
Black, non-Hispanic	1,127	24.5	(20.7–28.3)	14,338	15.3	(14.3–16.2)	<0.001
Other	700	18.2	(13.6–22.8)	7,822	12.1	(10.9–13.4)	0.013
**Education**							
<High school diploma/GED	1,782	23.9	(20.2–27.6)	11,315	26.5	(24.8–28.2)	0.209
High school diploma/GED	3,106	32.1	(29.1–35.1)	29,794	27.2	(26.2–28.3)	0.003
>High school diploma/GED	4,392	22.2	(19.8–24.5)	88,608	12.8	(12.4–13.3)	<0.001
Unknown	66	—¶	—	397	16.4	(9.4–23.4)	0.199
**Annual family Income**							
$0–$34,999	4,205	30.9	(28.3–33.5)	26,629	26.0	(25.0–27.0)	0.001
$35,000–$74,999	2,924	24.1	(21.2–26.9)	41,491	19.5	(17.8–20.2)	0.002
≥$75,000	1,891	17.8	(13.9–21.7)	55,358	11.9	(11.2–12.6)	0.004
Unknown	325	23.9	(13.2–34.6)	6,637	14.0	(12.2–15.9)	0.073
**Poverty status** **							
Poor	1,557	29.6	(25.5–33.8)	8,047	26.7	(24.8–28.7)	0.207
Near poor	2,299	27.6	(24.3–31.0)	15,714	24.2	(22.8–25.5)	0.055
Not poor	4,812	23.7	(21.4–26.1)	98,495	15.5	(15.0–16.0)	<0.001
Unknown	677	26.4	(19.7–33.0)	7,858	17.1	(15.2–19.0)	0.008
**Health insurance coverage**							
Insured	5,548	23.7	(21.6–25.7)	109,501	15.2	(14.7–15.7)	0.732
Not insured	3,725	29.3	(26.7–31.9)	20,202	29.7	(27.5–30.0)	<0.001
Unknown	72	—	—	412	21.7	(13.1–30.2)	—
**U.S. Census Region** ††							
Northeast	1,549	26.7	(21.9–31.6)	23,777	15.8	(14.8–16.8)	<0.001
Midwest	2,085	30.7	(27.3–34.2)	30,935	20.5	(19.5–21.5)	<0.001
South	3,445	27.8	(25.4–30.1)	46,060	18.4	(17.6–19.3)	<0.001
West	2,266	18.0	(15.2–20.9)	29,342	13.5	(12.8–14.2)	0.003

**Abbreviations:** CI = confidence interval; GED = General Educational Development certificate.

* Reported having smoked ≥100 cigarettes during their lifetime and currently smoking every day or some days.

† Two-tailed t-tests were used to determine statistically significant differences between smoking among accommodation and food services workers with all other workers combined. Additional information available at http://www.cdc.gov/nchs/data/series/sr_10/sr10_256.pdf.

§ Estimated average annual number of adults who were employed during the week before interview. Estimated total number of working adults is rounded down to the nearest 1,000.

¶ Estimates suppressed because relative standard error for estimate was >30%.

** Poverty status is based on family income and family size using the U.S. Census Bureau’s poverty thresholds for the previous calendar year. “Poor” persons are defined as being below the poverty threshold. “Near poor” persons have family incomes of 100% to <200% of the poverty threshold. “Not poor” persons have family incomes that are ≥200% of the poverty threshold. Additional information available at ftp://ftp.cdc.gov/pub/health_statistics/nchs/dataset_documentation/nhis/2008/srvydesc.pdf.

†† Additional information available at http://www2.census.gov/geo/pdfs/maps-data/maps/reference/us_regdiv.pdf.

**TABLE 2:** Characteristics of cigarette smokers among adults aged ≥18 years currently working in accommodation and food services sector — National Health Interview Survey, 2011–2013

	Accommodation and food services sector workers		All other workers (excluding accommodation and food services sector workers)		
			
Characteristic	%	(95% CI)	%	(95% CI)	p value*
**Education**					
<High school diploma/GED	17.7	(15.0–20.4)	13.3	(12.4–14.2)	0.003
High school diploma/GED	41.2	(37.4–45.0)	36.0	(34.7–37.4)	0.011
>High school diploma/GED	40.2	(36.3–44.0)	50.4	(49.0–51.8)	<0.001
**Poverty status** †					
Poor	22.3	(19.4–25.3)	11.6	(10.8–12.5)	<0.001
Near poor	28.4	(24.8–32.2)	19.0	(17. 9–20.2)	<0.001
Not poor	41.4	(37.1–45.7)	62.9	(61.6–64.4)	<0.001
**Frequency of smoking**					
Every day	76.4	(73.1–79.7)	75.4	(74.3–76.5)	0.583
Some days	23.6	(20.3–26.9)	24.6	(23.5–25.7)	0.583
**No. of cigarettes per day**					
≤12	70.6	(66.5–74.7)	61.6	(61.0–63.8)	<0.001
>12	29.4	(25.3–33.6)	38.4	(36.2–39.0)	<0.001
**Attempted to quit smoking** §					
Yes	48.0	(43.9–52.1)	46.5	(45.2–47.8)	0.503
No	52.0	(47.9–56.1)	53.5	(52.2–54.8)	0.503
**Years on the job**					
≤5	75.5	(71.8–79.2)	58.3	(56.9–59.7)	<0.001
>5	24.5	(20.8–28.2)	41.7	(40.3–43.1)	<0.001
**Self-rated physical health** ¶					
Excellent/Good	91.3	(89.2–93.5)	90.9	(90.2–91.6)	0.413
Poor/Fair	8.7	(6.5–10.9)	9.1	(8.4–9.8)	0.413
**Self-rated emotional health** **					
Poor	63.3	(57.0–69.6)	59.8	(57.6–62.0)	0.300
Excellent/Good	36.7	(30.5–43.0)	40.2	(38.0–42.4)	0.300

**Abbreviations:** CI = confidence interval; GED = General Educational Development certificate.

* Two-tailed t-tests were used to determine statistically significant differences between smoking among accommodation and food services workers with all other workers combined. Additional information available at http://www.cdc.gov/nchs/data/series/sr_10/sr10_256.pdf.

† Poverty status is based on family income and family size using the U.S. Census Bureau’s poverty thresholds for the previous calendar year. “Poor” persons are defined as being below the poverty threshold. “Near poor” persons have family incomes of 100% to <200% of the poverty threshold. “Not poor” persons have family incomes that are ≥200% of the poverty threshold. Additional information available at ftp://ftp.cdc.gov/pub/health_statistics/nchs/dataset_documentation/nhis/2008/srvydesc.pdf.

§ Attempts to quit smoking were based on responses to the question, “During the past 12 months, have you stopped smoking for more than 1 day because you were trying to quit smoking?”

¶ Physical health was based on the responses to the question, “Would you say your health in general is excellent, good, fair, or poor?”

** Emotional health was based on the responses to the question, “Have you felt sad, nervous, restless or fidgety, hopeless, that everything was an effort, or worthless, in the past 30 days?

**TABLE 3:** Cigarette smoking* prevalence among adults aged ≥18 years working in accommodation and food services sector, by industry and occupation — National Health Interview Survey, 2011–2013

Industry and occupation	Estimated population (in thousands)†	Smoking prevalence (%)	(95% CI)
**Industry**			
Food services and drinking places	7,899	26.8	(25.1–28.6)
Accommodation	1,446	20.6	(17.0–24.2)
**Occupation**			
Motor vehicle operators and material moving and other transportation	241	37.2	(23.6–50.8)
Management	1,170	32.6	(27.5–37.6)
Supervisors, food preparation	711	27.3	(21.4–33.2)
Food and beverage serving	2,589	27.0	(23.7–30.3)
Cooks and food preparation	1,952	26.5	(22.7–30.3)
Building and ground cleaning and maintenance	567	20.7	(14.5–27.0)
Other food preparation and serving related	557	17.8	(11.8–23.8)
Office and administrative support	419	17.3	(11.1–23.5)
Sales and related	692	17.0	(11.3–22.8)
Other food service workers	416	30.9	(21.5–40.3)
Unknown	30	—§	—

* Persons who reported smoking ≥100 cigarettes during their lifetime and who at the time of interview reported smoking every day or some days.

† Estimated annual average number of adults who were employed during the week before interview. Total number of working adults is rounded down to the nearest 1,000.

§ Estimates suppressed because relative standard error for estimate was >30%.

## Footnotes

* Additional information available at http://www.cdc.gov/nchs/data/series/sr_10/sr10_256.pdf.

† Accommodation and food services sector comprises establishments providing customers with lodging and/or preparing meals, snacks, and beverages for immediate consumption, and includes two industries: accommodation and food serving and drinking places. Additional information available at ftp://ftp.cdc.gov/pub/Health_Statistics/NCHS/Datasets/NHIS/NAICS_Sectors_and_Subsectors.pdf.

§ Additional information on cessation interventions for workers available at http://www.cdc.gov/niosh/docs/2015-113.

¶ Additional information available at http://www.cdc.gov/niosh/twh.
